# Comment on Altobelli et al. Colon Capsule Endoscopy as a Promising Diagnostic Tool in Colorectal Cancer: A Systematic Review and Network Meta-Analysis. *Diagnostics* 2025, *15*, 2157

**DOI:** 10.3390/diagnostics15232938

**Published:** 2025-11-21

**Authors:** Ian Io Lei, Ramesh Arasaradnam, Wojciech Marlicz, Anastasios Koulaouzidis

**Affiliations:** 1 Institute of Precision Diagnostics & Translational Medicine, University Hospital of Coventry and Warwickshire, Clifford Bridge Rd, Coventry CV2 2DX, UK; r.arasaradnam@warwick.ac.uk; 2 Warwick Medical School, University of Warwick, Coventry CV4 7AL, UK; 3 Leicester Cancer Centre, University of Leicester, Leicester LE1 7RH, UK; 4 Department of Gastroenterology, Pomeranian Medical University, 70-204 Szczecin, Poland; wojciech.marlicz@sanprobi.pl (W.M.); akoulaouzidis@hotmail.com (A.K.); 5 Department of Clinical Research, University of Southern Denmark, 5230 Odense, Denmark; 6 Department of Surgery, Odense University Hospital, 5000 Odense, Denmark

## 1. Introduction

Altobelli et al.’s systematic review and network meta-analysis (NMA) [1] provides a timely contribution to the evolving landscape of non-invasive colorectal cancer (CRC) screening. By comparing the diagnostic accuracy (sensitivity [sens] 0.75–0.79, specificity [spec] 0.95) of colon capsule endoscopy (CCE) against colonoscopy (COL) and computed tomographic colonography (CTC), stratified by screening levels (first level vs. second level), the study reinforces CCE as a promising alternative for patients averse to invasive procedures. Conducting such an analysis on diagnostic accuracy data is a complex task, and their effort significantly advances methodological and clinical understanding in this evolving domain. This is particularly significant as healthcare systems work to recover from post-COVID endoscopy backlogs while facing increasing colorectal cancer incidence. However, as evidenced by a contemporaneous umbrella meta-review by Lei et al. [2], refinements could enhance precision and applicability. Herein, we outline methodological considerations, results enhancements, broader implications, and a call for integration, while noting the absence of truly negative studies that might challenge prevailing positive evidence with implementation challenges in real-world screening settings [3].

## 2. Methodological Considerations

Altobelli et al. employed a robust search strategy across four databases (up to 14 April 2025), yielding 14 primary studies (*n* = 3088) assessed via QUADAS-2 (low-moderate risk of bias) [1]. Their random-effects meta-analysis and frequentist NMA innovatively address the lack of direct CCE–COL comparisons, ranking COL highest (P-score 0.999), followed by CCE (0.199) and CTC (0.001) [1]. The authors justified the NMA on the basis that no direct comparisons between CCE and colonoscopy (COL) were available, suggesting that only indirect evidence could inform relative performance. However, several of the included studies, such as Rex et al. (2015) [4], Parodi et al. (2018) [5], Voska et al. (2019) [6], Spada et al. (2012) [7], and Hagel et al. (2014) [8], were designed as paired accuracy trials in which all patients underwent both CCE and COL. This provides direct head-to-head diagnostic accuracy data. We acknowledge that these direct head-to-head studies are often limited in scope and vary across populations with different frameworks. The use of NMA, therefore, aimed to synthesise both direct and indirect evidence across a broader network of diagnostic strategies to yield a more comprehensive comparative assessment of CCE’s performance. Recognising this rationale, it remains important to clearly distinguish between the absence of large-scale comparative effectiveness trials and the availability of paired accuracy data. Clarifying this distinction would enhance transparency and strengthen the methodological justification for employing NMA as a means of integrating evidence from diverse study designs.

The validity of any NMA depends fundamentally on the assumption of transitivity, that studies are sufficiently comparable in terms of populations, disease spectrum, and diagnostic thresholds to justify indirect comparisons. If transitivity holds, one can assume that differences in observed effects arise from the tests themselves rather than from differences between studies. While transitivity cannot be verified statistically, its empirical manifestation, consistency, can be evaluated when closed loops of evidence exist within the network. In such settings, node-splitting or design-by-treatment interaction models can be applied to assess the statistical agreement between direct and indirect estimates, as described by Salanti (2012) [9].

In addition, the application of standardised mean differences (SMDs) in this diagnostic NMA is methodologically atypical, a point acknowledged by the authors in the study’s limitations. Sensitivity and specificity are inherently correlated and threshold-dependent, treating them as continuous outcomes risks misrepresenting diagnostic accuracy. Established approaches, such as bivariate generalised linear mixed models or hierarchical summary receiver operating characteristic (HSROC) curves, would enable the assessment of global metrics, including the area under the curve (AUC). However, we acknowledge that extending these approaches to a network meta-analysis framework remains statistically challenging. Although several Bayesian adaptations of bivariate and HSROC models have been proposed [10,11], a universally accepted “gold-standard” model for diagnostic test accuracy NMAs has yet to be established.

High heterogeneity (I^2^ up to 99.7%) is acknowledged and attributed to bowel preparation variability (e.g., diverse polyethylene glycol (PEG)-based protocols with boosters like sodium phosphate or prucalopride, as detailed in Table 3) [1]. However, potential additional sources of heterogeneity, including preparation adequacy (40–90% across studies) and capsule completion rates (64–100%), were not further presented through meta-regression or sensitivity analyses. These added factors might have provided additional insight into study-level differences. Lei et al.’s restricted maximum likelihood (REML) meta-regression on similar covariates found no significant associations but quantified their influence, reducing I^2^ to <25% in subgroups [2]. Applying similar exploratory analyses could further strengthen the interpretability of Altobelli et al.’s findings. Additionally, certain limitations in the search strategy warrant consideration. Although multiple databases were queried, several CCE–COL trials identified in Lei et al.’s meta-review were not captured. This may reflect the use of relatively narrow keyword combinations, omitting terms such as “video capsule colonoscopy” or “PillCam”, along with limited MeSH/Emtree expansion and the absence of backward citation searching.

## 3. Results and Evidence Integration

Altobelli et al.’s pooled estimates (first-level sensitivity 0.79 [95% CI: 0.60–0.91], specificity 0.95; second-level sensitivity 0.75 [0.65–0.83], specificity 0.95) converge with Lei et al.’s overall figures (any polyps sens 0.79 [0.69–0.86], spec 0.77, AUC 0.81) [1,2], affirming consistency. Both demonstrate improved accuracy for larger polyps (>9 mm in Altobelli: sensitivity 0.85–0.95, specificity 0.97–0.98; ≥10 mm in Lei: sensitivity 0.88, specificity 0.95, AUC 0.95) [1,2], highlighting the utility of CCE for clinically relevant lesions that warrant polypectomy and surveillance. Lei quantifies CRC sensitivity at 0.96 [0.73–1.00] after excluding incompletes (e.g., battery exhaustion, a common limitation in primaries like Van Gossum et al., 2009) [2]. Incorporating CRC-specific metrics could potentially enrich Altobelli et al.’s clinical guidance.

To assess evidence robustness, we conducted a web search (02:31 PM EEST, 29 August 2025) for “negative studies or criticisms on CCE diagnostic accuracy 2025,” yielding 15 results [12,13,14,15,16,17,18,19,20,21,22,23]. Predominantly affirmative or neutral, the literature emphasises limitations like variable excretion rates (57–100%) [14] and reader fatigue (accuracy decline post-first study) [16], but lacks truly negative studies discrediting CCE’s comparability to COL. Criticisms often target reference standard imperfections (e.g., COL interval CRC rates biasing against CCE) [3] or follow-up needs (e.g., conversion factors) [8]. A notable exception is the large Randomised Controlled Trial (RCT) by Baatrup et al. [3], which found that offering CCE as an alternative to colonoscopy in FIT-positive patients did not improve uptake (91.7% vs. 91.1%) or advanced neoplasia detection (0.67% vs. 0.64%), while resulting in a 70% secondary colonoscopy rate due to incomplete CCE or positive findings. This scarcity of negative trials implies that only rigorously designed, large-scale studies demonstrating inferior outcomes would meaningfully shift the prevailing evidence base, which remains strongly supportive of CCE. At the same time, innovations in service delivery may alter uptake. The introduction of home-based CCE delivery, potentially facilitated by remote monitoring and 5G connectivity, could make participation more convenient and accessible [12,24]. Robust large-scale evaluations of home-delivered CCE are now required, assessing not only completion rates and need for reinvestigation but also patient experience, which may prove decisive in shaping future adoption.

Other evidence gaps need to be addressed; future searches should explicitly target critical perspectives, for example, “CCE limitations in diverse populations” or “cost-effectiveness critiques of CCE” [4,5,6,7,8,9,10,11,12,13,14,15,16,17,18,19]. As Altobelli et al. note, appropriate patient selection is central to reducing the need for secondary colonoscopy by improving completion rates, bowel preparation quality, and pathology yield [25,26,27]. Indication-specific use also warrants attention; for instance, applying panenteric capsule endoscopy in cases of iron deficiency anaemia may obviate the need for a subsequent small-bowel capsule procedure, thereby enhancing cost-effectiveness [28]. Economic evaluations should also incorporate emerging data from ongoing trials such as the CESCAIL study, which explores AI-assisted CCE to optimise accuracy and efficiency [16,29].

## 4. Broader Implications

**Figure 1:**
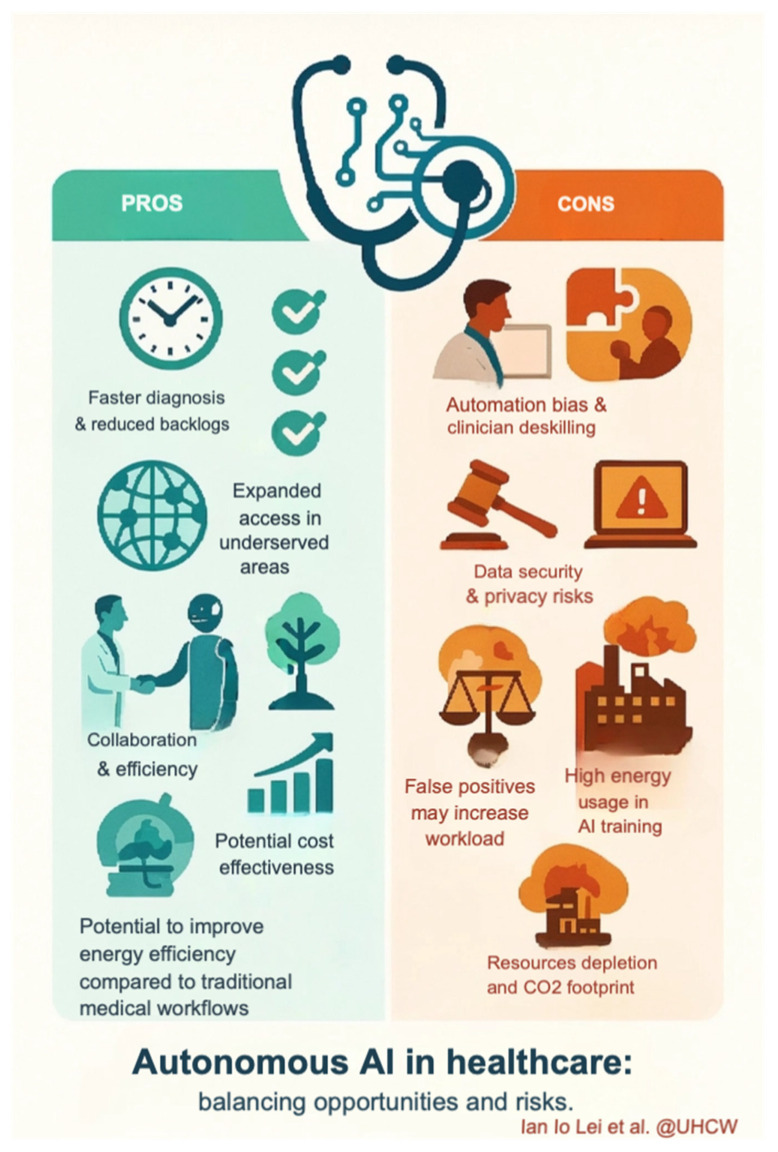
The Pros and Cons of Autonomous AI in Healthcare.

Altobelli et al. appropriately highlight CCE’s role for patients refusing colonoscopy, citing cost-effectiveness analyses from the UK and Scotland showing per-procedure costs of £747 versus £900 [1]. Lei et al. expand this perspective, noting both the rarity of complications such as capsule retention and potential savings in symptomatic settings, with £6.71 per patient reported from National Services Scotland micro-costing data [2]. As emphasised above, follow-up endoscopy rates should be considered as key indicators of cost-effectiveness [2,13]. By contrast, findings from Baatrup et al. suggest that in FIT-based screening programmes with high baseline adherence, CCE may not reduce system burden and could increase costs due to frequent reinvestigations [3]. Careful patient selection with integrated artificial intelligence (AI) could mitigate these challenges: tools such as AiSPEED™, currently under evaluation in the CESCAIL study [2,17], may reduce reader variability and improve scalability. The application of AI also raises issues of equity and sustainability. While AI has the potential to expand access in low-resource settings, the computational demands of training and deployment contribute to carbon emissions [30,31]. Conversely, autonomous AI systems, where medical decisions are made without human oversight, could reduce greenhouse gas emissions by up to 80% compared with clinician-led systems [32]. Future research should therefore incorporate environmental impact into evaluations of telemedicine and AI applications in CCE [12] (see Figure 1).

## 5. Conclusions and Call to Action

Altobelli et al.’s study represents a substantial and technically demanding contribution to the evidence base on CCE. Conducting an NMA on diagnostic test accuracy data is inherently complex, and this work significantly advances comparative evaluation across multiple CRC screening modalities. Their analysis complements Lei et al.’s recent synthesis of diagnostic accuracy [1,2]. Future analyses incorporating bivariate or hierarchical models, generational distinctions, and CRC-specific outcomes could yield more nuanced and clinically applicable insights. In the absence of rigorously negative studies, the diagnostic promise of CCE appears strong and supports consideration for guideline integration, including by ESGE [33]. Nevertheless, pragmatic trials such as those by Baatrup et al. [3] emphasise that implementation within FIT-positive cohorts may not be cost-effective unless reinvestigation rates can be reduced. A collaborative update combining these methodological refinements, pragmatic trial evidence, and evolving technologies is warranted to optimise the role of CCE in CRC screening strategies.

## References

[1] Altobelli E., Angeletti P.M., Varesini P.A., Bianchi Z., Masedu F. (2025). Colon Capsule Endoscopy as a Promising Diagnostic Tool in Colorectal Cancer: A Systematic Review and Network Meta-Analysis. Diagnostics.

[2] Lei I.I., Cortegoso Valdivia P., Marlicz W., Skonieczna-Zydecka K., Arasaradnam R., Eliakim R., Koulaouzidis A. (2025). Systematic meta-review: Diagnostic accuracy of colon capsule endoscopy for colonic neoplasia with umbrella meta-analysis. Ther. Adv. Gastrointest. Endosc..

[3] Baatrup G., Bjorsum-Meyer T., Kaalby L., Schelde-Olesen B., Kobaek-Larsen M., Koulaouzidis A., Kroijer R., Al-Najami I., Buch N., Hogh A. (2025). Choice of colon capsule or colonoscopy versus default colonoscopy in FIT positive patients in the Danish screening programme: A parallel group randomised controlled trial. Gut.

[4] Rex D.K., Adler S.N., Aisenberg J., Burch W.C., Carretero C., Chowers Y., Fein S.A., Fern S.E., Fernandez-Urien Sainz I., Fich A. (2015). Accuracy of capsule colonoscopy in detecting colorectal polyps in a screening population. Gastroenterology.

[5] Parodi A., Vanbiervliet G., Hassan C., Hebuterne X., De Ceglie A., Filiberti R.A., Spada C., Conio M. (2018). Colon capsule endoscopy to screen for colorectal neoplasia in those with family histories of colorectal cancer. Gastrointest. Endosc..

[6] Voska M., Grega T., Ngo O., Buckova B., Majek O., Vojtechova G., Tacheci I., Benes M., Bures J., Spicak J. (2019). The comparison of the efficiency of colon capsule endoscopy and optical colonoscopy in patients with positive immunochemical fecal occult blood test: Multicenter, prospective study. Endoscopy.

[7] Spada C., De Vincentis F., Cesaro P., Hassan C., Riccioni M.E., Grazioli L.M., Bolivar S., Zurita A., Costamagna G. (2012). Accuracy and safety of second-generation PillCam COLON capsule for colorectal polyp detection. Ther. Adv. Gastroenterol..

[8] Hagel A.F., Gabele E., Raithel M., Hagel W.H., Albrecht H., De Rossi T.M., Singer C., Schneider T., Neurath M.F., Farnbacher M.J. (2014). Colon capsule endoscopy: Detection of colonic polyps compared with conventional colonoscopy and visualization of extracolonic pathologies. Can. J. Gastroenterol. Hepatol..

[9] Salanti G. (2012). Indirect and mixed-treatment comparison, network, or multiple-treatments meta-analysis: Many names, many benefits, many concerns for the next generation evidence synthesis tool. Res. Synth. Methods.

[10] Ma X., Lian Q., Chu H., Ibrahim J.G., Chen Y. (2018). A Bayesian hierarchical model for network meta-analysis of multiple diagnostic tests. Biostatistics.

[11] Owen R.K., Cooper N.J., Quinn T.J., Lees R., Sutton A.J. (2018). Network meta-analysis of diagnostic test accuracy studies identifies and ranks the optimal diagnostic tests and thresholds for health care policy and decision-making. J. Clin. Epidemiol..

[12] Jalayeri Nia G., Conway C., Ward F., Dungey S., Streames L., Liu B.B., Lei I.L., Cameron J., Wenzek H., Shekhar C. (2024). Exploring the feasibility of home-delivered capsule endoscopy with 5G support: Innovations and carbon footprint insights. BMJ Open Gastroenterol..

[13] Bjoersum-Meyer T., Skonieczna-Zydecka K., Cortegoso Valdivia P., Stenfors I., Lyutakov I., Rondonotti E., Pennazio M., Marlicz W., Baatrup G., Koulaouzidis A. (2021). Efficacy of bowel preparation regimens for colon capsule endoscopy: A systematic review and meta-analysis. Endosc. Int. Open.

[14] Spada C., Pasha S.F., Gross S.A., Leighton J.A., Schnoll-Sussman F., Correale L., Gonzalez Suarez B., Costamagna G., Hassan C. (2016). Accuracy of First- and Second-Generation Colon Capsules in Endoscopic Detection of Colorectal Polyps: A Systematic Review and Meta-analysis. Clin. Gastroenterol. Hepatol..

[15] Lei I.I., Agache A., Robertson A., Thorndal C., Deding U., Arasaradnam R., Koulaouzidis A. (2025). Follow-up endoscopy rates as an indicator of effectiveness in colon capsule endoscopy: A systematic review and meta-analysis. BMJ Open Gastroenterol..

[16] Lei I.I., Tompkins K., White E., Watson A., Parsons N., Noufaily A., Segui S., Wenzek H., Badreldin R., Conlin A. (2023). Study of capsule endoscopy delivery at scale through enhanced artificial intelligence-enabled analysis (the CESCAIL study). Colorectal Dis..

[17] Nadimi E.S., Braun J.M., Schelde-Olesen B., Khare S., Gogineni V.C., Blanes-Vidal V., Baatrup G. (2025). Towards full integration of explainable artificial intelligence in colon capsule endoscopy’s pathway. Sci. Rep..

[18] Morgan E., Arnold M., Gini A., Lorenzoni V., Cabasag C.J., Laversanne M., Vignat J., Ferlay J., Murphy N., Bray F. (2023). Global burden of colorectal cancer in 2020 and 2040: Incidence and mortality estimates from GLOBOCAN. Gut.

[19] Vuik F.E.R., Nieuwenburg S.A.V., Moen S., Spada C., Senore C., Hassan C., Pennazio M., Rondonotti E., Pecere S., Kuipers E.J. (2021). Colon capsule endoscopy in colorectal cancer screening: A systematic review. Endoscopy.

[20] Kjolhede T., Olholm A.M., Kaalby L., Kidholm K., Qvist N., Baatrup G. (2021). Diagnostic accuracy of capsule endoscopy compared with colonoscopy for polyp detection: Systematic review and meta-analyses. Endoscopy.

[21] Alihosseini S., Sabermahany A., Aryankhesal A. (2021). Second-generation colon capsule endoscopy for detection of colorectal polyps: Systematic review. Color. Dis..

[22] Mollers T., Schwab M., Gildein L., Hoffmeister M., Albert J., Brenner H., Jager S. (2021). Second-generation colon capsule endoscopy for detection of colorectal polyps: Systematic review and meta-analysis of clinical trials. Endosc. Int. Open.

[23] Sulbaran M., Bernardo W.M., Bustamante-Lopez L.A., Sakai C.M., Sakai P., Nahas S.C., De Moura E.G. (2021). Systematic review and meta-analysis of colon capsule endoscopy accuracy for colorectal cancer screening. An alternative during the covid era?. Gastrointest. Endosc..

[24] Parisi I., Hosea A.V., Stoffel S., Nemec M., Badat S., Seward E., Kaushal A., Kerrison R., Von Wagner C. (2024). Evaluation of the safety, efficacy and feasibility of ‘at-home’ capsule endoscopy. Frontline Gastroenterol..

[25] Lei I.I., Parisi I., Bhandare A., Perez F.P., Lee T., Shehkar C., McStay M., Anderson S., Watson A., Conlin A. (2025). Factors predicting conversion from colon capsule endoscopy to conventional optical endoscopy-findings from the CESCAIL study. BMC Gastroenterol..

[26] MacLeod C., Foxton A., Wilson P., Treweek S., Watson A.J.M. (2023). Associations between patient factors and successful colon capsule endoscopy—A prospective cohort study. Color. Dis..

[27] Moen S., Vuik F.E.R., Voortman T., Kuipers E.J., Spaander M.C.W. (2022). Predictors of Gastrointestinal Transit Times in Colon Capsule Endoscopy. Clin. Transl. Gastroenterol..

[28] Lei I.I., O’Connell N., Adu-Darko M.A., Parambil J., Suresh V., Mc Donnell K., Newville J., Chaplin K., Siyambalapityage D., Khan A. (2025). From Stool to Scope: Optimising FIT Thresholds to Guide Future Panenteric Capsule Endoscopy and Reduce Colonoscopy Burden in Iron Deficiency Anaemia. Cancers.

[29] Lei I.I., Parsons N., Huhulea C., Wenzek H., White E., Laiz P., Noble C., Robertson A., Koulaouzidis A., Arasaradnam R. (2025). Diagnostic accuracy of CADe-assisted reading versus clinician reading for polyp detection in colon capsule endoscopy: A multicentre prospective study. Clin. Med..

[30] Truhn D., Muller-Franzes G., Kather J.N. (2024). The ecological footprint of medical AI. Eur. Radiol..

[31] Ding Z., Wang J., Song Y., Zheng X., He G., Chen X., Zhang T., Lee W.J., Song J. (2025). Tracking the carbon footprint of global generative artificial intelligence. Innovation.

[32] Wolf R.M., Abramoff M.D., Channa R., Tava C., Clarida W., Lehmann H.P. (2022). Potential reduction in healthcare carbon footprint by autonomous artificial intelligence. NPJ Digit. Med..

[33] Tawfik G.M., Giang H.T.N., Ghozy S., Altibi A.M., Kandil H., Le H.H., Eid P.S., Radwan I., Makram O.M., Hien T.T.T. (2020). Protocol registration issues of systematic review and meta-analysis studies: A survey of global researchers. BMC Med. Res. Methodol..

